# Proprioceptive Neuromuscular Facilitation (PNF): Its Mechanisms and Effects on Range of Motion and Muscular Function

**DOI:** 10.2478/v10078-012-0011-y

**Published:** 2012-04-03

**Authors:** Kayla B. Hindle, Tyler J. Whitcomb, Wyatt O. Briggs, Junggi Hong

**Affiliations:** 1 Exercise Science Department, Willamette University, Salem, Oregon, USA.

**Keywords:** athletic performance, gender differences, age differences, stretching

## Abstract

Proprioceptive neuromuscular facilitation (PNF) is common practice for increasing range of motion, though little research has been done to evaluate theories behind it. The purpose of this study was to review possible mechanisms, proposed theories, and physiological changes that occur due to proprioceptive neuromuscular facilitation techniques. Four theoretical mechanisms were identified: autogenic inhibition, reciprocal inhibition, stress relaxation, and the gate control theory. The studies suggest that a combination of these four mechanisms enhance range of motion. When completed prior to exercise, proprioceptive neuromuscular facilitation decreases performance in maximal effort exercises. When this stretching technique is performed consistently and post exercise, it increases athletic performance, along with range of motion. Little investigation has been done regarding the theoretical mechanisms of proprioceptive neuromuscular facilitation, though four mechanisms were identified from the literature. As stated, the main goal of proprioceptive neuromuscular facilitation is to increase range of motion and performance. Studies found both of these to be true when completed under the correct conditions. These mechanisms were found to be plausible; however, further investigation needs to be conducted. All four mechanisms behind the stretching technique explain the reasoning behind the increase in range of motion, as well as in strength and athletic performance. Proprioceptive neuromuscular facilitation shows potential benefits if performed correctly and consistently.

## Introduction

Proprioceptive Neuromuscular Facilitation (PNF) is a stretching technique utilized to improve muscle elasticity and has been shown to have a positive effect on active and passive range of motions (Funk et al., 2003; Lucas and Koslow, 1984; Wallin et al., 1985). Recent research has been focused on the efficacy of the intervention on certain outcome measures, such as passive range of motion (PROM), active range of motion (AROM), peak torque and muscular strength. This review is important for the justification of its usage within therapeutic and athletic settings in order to rehabilitate injuries by gaining AROM and PROM or improving performance. In clinical settings, PNF is already utilized by therapists to restore functional range of motion (ROM) and increase strength in patients who have sustained soft tissue damage or received invasive surgeries.

Currently, research has proven that PNF techniques do increase ROM (Funk et al., 2003; Lucas and Koslow, 1984; Wallin et al., 1985). Two techniques are seen in the literature more frequently than others, the contract-relax method (CR) and the contract-relax-antagonist-contract method (CRAC) of PNF. The CR method included the target muscle (TM) being lengthened and held in that position while the participant contracted the TM to its maximum isometrically for an allotted amount of time. This was followed by a shorter relaxation of the TM that usually included a passive stretch (Etnyre and Abraham, 1986). The CRAC method followed the exact same procedure as the CR method, but was continued further. Instead of just passively stretching the TM, the participant contracted the antagonist muscle to the TM for another allotted period of time (Etnyre and Abraham, 1986). PNF has also been found to increase muscular performance when performed in regard to exercise. If performed before exercise, it will actually decrease muscular performance; however, studies have shown that if PNF is performed either after or without exercise it increases muscular performance (Bradley et al., 2007; Marek et al., 2005; Mikolajec et al., 2012; Nelson et al., 1986). In order to maintain these increases, both for ROM and muscular performance, it necessary to do at least two sets of PNF each week.

Research behind stretching has been relatively inconclusive in examining the effects of static stretching (SS), ballistic stretching (BS), and PNF stretching on outcome measures, such as injury prevention and athletic performance. The only noted difference between the three stretching protocols has been PNF’s ability to cause a larger magnitude of gains within subjects’ ROM, both active and passive (Funk et al., 2003; Lucas and Koslow, 1984; Wallin et al., 1985; Etnyre and Lee, 1988; Feland et al., 2001). There are almost no physiological mechanisms that lead to an increase in ROM proposed in the literature. The four theoretical mechanisms discussed in the literature will be further discussed in this review. These four mechanisms are: autogenic inhibition, reciprocal inhibition, stress relaxation, and the gate control theory (all of which provide potential ways for PNF to increase ROM) (Sharman et al., 2006; Rowlands et al., 2003). PNF has been compared to the traditional methods of stretching (SS and BS) when it comes to ROM, athletic performance, and power output (Funk et al., 2003; Lucas and Koslow, 1984; Etnyre and Lee, 1988; Feland et al., 2001). However, its effect on muscular function is less clear, as it decreases muscular function when performed before exercise yet increases it when performed afterward (Bradley et al., 2007; Marek et al., 2005; Mikolajec et al., 2012; Nelson et al., 1986). This effect on muscular function is discussed in this review paper. Other factors that can affect the desired effects of PNF include, the age and gender of the person PNF is being performed on, the duration of the contraction, the specific muscles being stretched, the technique employed (CR or CRAC), and the percentage of the maximal voluntary isometric contraction (MVIC) performed. Only a few studies that were found discussed these other factors (Etnyre and Lee, 1988; Feland et al., 2001; Feland and Marin, 2004; Rowlands et al., 2003). These studies are discussed briefly in this paper, but more research into why these factors affect the outcomes of PNF is necessary in order to provide more useful information for the use of PNF as a rehabilitation technique in a clinical setting.

In order to accurately and effectively treat patients, therapist and trainers use Evidence Based Sports Medicine, in which rehabilitation protocols are designed using techniques validated through scientific research and thoroughly understood by the scientific community. Even so, PNF is used consistently without an understanding of the basic elements of how it works (even though studies have delved into the effectiveness of techniques of PNF and compared it to other forms of stretching.) Although, the results of the studies used in this review were conclusive when discussing the changes in ROM and muscular performance, the literature did not discuss the theoretical mechanisms behind PNF. If the studies used did mention a theoretical mechanism, the discussions were brief. Few studies even mentioned the underlying mechanisms that cause the physiological changes within the body as a result of PNF. Only one study provided an attempt to evaluate the theories behind PNF stretching. Thus, there has been no previous systemic review that intensely examined the proposed theories and the physiological changes occurring during PNF stretching that would result in the alterations in performance of certain outcome measures behind PNF stretching (Sharman et al., 2006). Therefore, this systematic review aims to break down the literature surrounding the physiological mechanisms and adaptations that occur during PNF stretching in order to provide a foundation upon which application of this technique will be validated, along with describing PNF as a means of gaining more ROM and helping to develop muscular strength and performance (Nelson et al., 1986).

### Theoretical Mechanisms

Four theoretical physiological mechanisms for increasing ROM were identified: autogenic inhibition, reciprocal inhibition, stress relaxation, and the gate control theory (Sharman et al., 2006; Rowlands et al., 2003). Each of these theoretical mechanisms are reflexes that occur when the Golgi tendon organs (GTOs) in the tendons of the TM, or in the antagonist muscle to the TM, detect harmful stimuli (such as a stretching sensation or during a contraction). Each theory can be used to explain why an increase in ROM during both the CR and CRAC methods of PNF discussed in this paper can occur.

### Autogenic Inhibition

Autogenic Inhibition is what occurs in a contracted or stretched muscle in the form of a decrease in the excitability because of inhibitory signals sent from the GTOs of the same muscle (Sharman et al., 2006). This tension causes activation of Ib afferent fibers within the GTOs. Afferent fibers send signals to the spinal cord where the stimulus causes the activation of inhibitory interneurons within the spinal cord. These interneurons place an inhibitory stimulus upon the alpha motoneuron, decreasing the nerves’ excitability and decreasing the muscles’ efferent motor drive (Sharman et al., 2006). It is theorized that this reflex occurs as the body attempts to spread the workload evenly across the motor unit within the muscle, assisting the asynchronous recruitment of the body in preventing specific motor units from fatiguing. This chain reaction causes the TM to relax, which is one of the driving theories behind the increased elongation of the muscle fibers during the CR and CRAC methods of PNF stretching.

Autogenic inhibition relies on the body’s self-regulatory mechanisms of the GTOs in order to protect structures. However, in the case of both CR and CRAC PNF stretching, contraction of the TM during stretching and contraction of the antagonist muscle (CRAC) take advantage of this mechanism to decrease muscle tension, allowing for elongation of the muscles fibers. This allows the CRAC method of PNF stretching to take advantage of the viscoelastic properties of the musclotendious units, allowing the muscle to “creep” and elongate, thus increasing the ROM of the subject. Although, there is uncertainty as to how much of a part GTOs play in PNF stretching, and the long term improvements seen in subjects as a result (Sharman et al., 2006). Research has shown that GTOs have a major role in inhibition of muscle fibers, but the duration, and even the activation of this inhibition, is questionable. Studies have shown that after contraction, the activation of the inhibitory neurons of the GTOs are low or nonexistent, showing that the inhibitory signal of the GTOs is weak after contraction (Laporte and Lloyd, 1952). Note that muscle activation usually indicates movement or exercise, in which case muscle inhibition would be counterproductive. More research needs to be done on muscle activation during PNF stretching and regarding the duration of the muscle’s inhibition after PNF stretching, before any conclusions can be made.

### Reciprocal Inhibition

Reciprocal inhibition is what occurs in the TM when the opposing muscle is contracted voluntarily in the form of decreased neural activity in the TM (Sharman, 2006). It occurs when an opposing muscle is contracted in order to maximize its contraction force, in this case, the TM relaxes. This relaxation of the TM is a result of the decrease in the neural activity, and the increase of inhibition of proprioceptive structures in the TM (Rowlands et al., 2003). Inhibition of the electrical activity in the stretched TM occurs due to the neurons’ continuation of firing in the TM, the contraction of the antagonist muscles would be resisted and diminished by the force of the TM continuing to receive signals to contract. On a spinal level, Ia afferent fibers enter the spinal cord and give off collateral branches that interact with interneurons in the spine, which then send signals to the alpha-motoneuron in the GTOs of the TM. The effect of this connection is inhibitory and causes relaxation of the TM (The Nervous Statement, 2003; Sharman et al., 2006).

The mechanism of PNF referred to above, is the way in which TM and its antagonist muscles work together. When one contracts, the other relaxes and is thus inhibited in order to prevent the muscles from working against one another (Neuroscience Online, 2011). This potentially explains part of what is happening during the CRAC method of PNF. In the CRAC method, the contracting muscle in the “antagonist-contract” portion of the technique, brings about this reflex and inhibits the TM. This inhibition of the TM, along with the shortening contraction of the antagonist muscle, allows the muscle fibers of the TM to elongate even further, creating a greater stretching force for the TM and producing a larger inhibitory influence on the TM (Etnyre and Abraham, 1986; Sharman et al., 2006). The interneuron that innervates the alpha-motoneuron, which synapses onto the TM, causes the neural activity in the TM to decrease and leads to more stretching of the TM (Rowlands et al., 2003). More research on how long this reflex affects the TM needs to be done in order to prove reciprocal inhibition is behind the effects of PNF stretching.

### Stress Relaxation

Stress relaxation is what occurs when the musculotendinous unit (MTU), which involves the muscles and the connected tendons, is under a constant stress (Sharman et al., 2006). Both muscles and tendons have viscoelastic properties in which they exhibit characteristics of both viscous and elastic materials. A viscoelastic material both resists shear flow and strain linearly when stress is applied and returns to the original form once the stress is removed from the MTU. As what was mentioned before, when the MTU falls under a constant stretch, a phenomenon known as “stress relaxation” occurs. This decreases the force generated by the viscous material when it resists the elongation stimulus that stretching causes within the MTU. Because the viscous material loses its ability to resist the stretch over time, the MTU slowly increases in length, a property that is referred to as “creep” of the MTU (Sharman et al., 2006). There is a limit to how far a muscle can “creep,” as the longer a MTU gets, the higher the passive torque (resistance of MTU to stretching) and the muscle’s stiffness become (Sharman et al., 2006). Though, as the stretch is held, the stress relaxation occurs and there is a decrease in the passive torque and muscle stiffness that lasts for a short period of time (Sharman et al., 2006). This is a protective mechanism to prevent muscle tearing and maintain a healthy relationship between the contractile units of the muscle sarcomere. When the CR method is utilized in PNF stretching, the contraction of the TM increases the tensile stress upon the MTU, encouraging the “creep” of the muscle fibers when in an elongated orientation. This is similar to the CRAC method, except for the fact that the contraction of the antagonist muscle applies more tensile force on the TM.

Out of the four theories, the passive properties of the MTU is most applicable throughout each theory, as the viscoelastic properties of the muscle tissue itself allow for the muscle to be stretched and elongated as a result of the inhibitory signals, without substantial damage to the tissue during stretching. In order for there to be an increase in ROM and flexibility, there needs to be an adaptation within the muscle. The stress relaxation phenomenon of viscoelastic materials allows the material to “creep” and slowly lengthen over time, but studies have shown that it is change in passive torque within the muscle that allows the lengthening. It is usually short lived, lasting anywhere from 80 seconds to an hour after PNF stretching (Magnusson et al., 1996). Thus, although it seems as the viscoelastic properties of the muscle do account directly for the increased ROM experienced after PNF stretching, more research is needed on longer term adaptations to muscle tissue as a result of stretching for conclusive results.

### The Gate Control Theory

The gate control theory is what occurs when two kinds of stimuli, such as pain and pressure, activate their respective receptors at the same time (Mazzullo, 1978). Peripheral pain receptors are connected to either un-myelinated or small myelinated afferent fibers while pressure receptors are connected to larger myelinated afferent nerve fibers. Each type of afferent fibers connect to the same interneurons in the spine, and because the pressure afferent fibers are larger and myelinated the pressure signals make it to the spine before the pain signals do when they are stimulated simultaneously (Mazzullo, 1978). The inhibition of the pain signals happens in the dorsal horn when the large fibers transmit signals (Melzack, 1993). In CR and CRAC, when the muscle is stretched beyond its active ROM, the participant is then told to resist against this stretch, and then the TM is stretched even further. A large force and stretch is produced in the elongated muscle when the participant resists the stretch. This large force is sensed as noxious stimuli, and is seen as potentially damaging, which invites the GTOs to activate in an effort to inhibit the force and prevent injury. As this process is repeated with a consistent protocol, the nociception, or cause of the amount of inhibition of the GTOs, decreases as it becomes more accustomed to increased muscle and tendon length, as well as increased force. The GTOs adapt and decrease inhibition, allowing the muscle to produce a greater amount of force; however, this may increase the risk of injury. With increased muscle length comes ability to produce greater force because of the length-tension relationship. With increased ROM, and decreased GTO inhibition, the muscle may be able to increase its strength and force production.

In both CR and CRAC PNF stretching, the gate control theory is a plausible mechanism in gaining the benefits of the technique. The gate control theory argues that when the muscle is stretched forcefully, past its natural ROM, the GTOs are activated in an attempt reduce injury. In PNF stretching, not only are the muscles and tendons stretched, they are also contracted at this elongated length, decreasing the nociception, or pain that is sensed that causes inhibition, produced by the GTOs. The GTOs adapt to the increase in length and force threshold, which allow for greater force production. Some evidence suggests that GTOs play no role in sensing force or inhibiting it (Chalmers, 2002). If true, gate control theory would be discredited, however further investigation is needed to prove or disprove this theory.

### Effects of PNF

PNF is a stretching technique utilized to increase ROM and flexibility. PNF increases ROM by increasing the length of the muscle and increasing neuromuscular efficiency. PNF stretching has been found to increase ROM in trained, as well as untrained, individuals. Effects can last 90 minutes or more after the stretching has been completed (Funk et al., 2003). The duration of these effects can vary because of various things, such as changes in the percentage of MVIC asked for and the duration of the contraction of the TM during PNF stretching (Feland and Marin, 2004; Rowlands et al., 2003). PNF stretching is usually performed with a 100% MVIC, which can possibly lead to of a contraction induced injury and/or muscle soreness. Lower percentages of MVIC might reduce these risks (Feland and Marin, 2004). This contraction has been proven to produce better effects when held a total of 3–10 seconds, while six seconds is preferred (Feland and Marin, 2004). It is necessary to know why six seconds is preferred and if there is any benefit to a longer or shorter contraction. There are also noticeable differences in ROM as a result of PNF found between genders and age groups (Etnyre and Lee, 1988; Feland et al., 2001). There is an increase in ROM and flexibility found regarding each variance, but to different degrees. Literature looking into each of these variations of PNF stretching, and just PNF stretching on ROM, are discussed further on. While there was a large amount of literature that solely looked at changes in ROM over time, or after one bout of PNF stretching, there was a limited amount found regarding the effects of the variations on ROM. This was also true in regard to the effects of PNF on athletic performance and muscular strength. Athletic performance was generally found to decrease when PNF stretching was performed before exercise, and increase when performed independent of exercise, or after exercise was completed (Marek et al., 2005; Mikolajec et al., 2012; Nelson et al., 1986). In general muscular strength has also been shown to increase due to PNF (Nelson et al., 1986). These two effects of PNF will also be discussed.

### Effects on Muscular Function

Stretching has long been viewed as beneficial to enhance performance and decrease risk of injury during exercise, as well as improve ROM and function following an injury (McCarthy et al., 1997). PNF stretching prior to exercise has been found to decrease performance when maximal muscle effort is required such as during sprinting, plyometrics, cutting, weight-lifting and other high intensity exercises (Bradley et al., 2007; Mikolajec et al., 2012). Marek et al. (2005) showed a decrease in strength, power output and muscle activation. Similar studies have shown a significant decrease in vertical jump height and power, as well as a decrease in ground reaction time and jump height, in drop jumps following PNF stretching (Bradley et al., 2007; Mikolajec et al., 2012).

Although PNF may decrease performance in high intensity exercises, it has been found to improve performance in submaximal exercises such as jogging. Caplan et al. (2009) showed a significant increase in both stride rate and stride length after a five week PNF stretching protocol in 18 professional rugby players. Nelson et al. (1986) showed PNF stretching to be similar in effectiveness to weight training in enhancing muscular strength; however, a significant increase in athletic performance in untrained females was determined as well. Vertical jump and throwing distance increased more than double in those in the PNF stretching group than those in the weight training group. The PNF group completed stretches twice a week for eight weeks. Each session consisted of three sets of six against maximal force on both lower and upper extremities. This study infers that PNF may enhance force production as well as functional movements in untrained individuals.

PNF stretching has been proven to decrease strength and power when done prior to high intensity and maximal effort exercises, such as jumping, plyometrics, sprinting, cutting, and other similar movements. These effects can last longer than ninety minutes. PNF is effective if completed after exercise and done at least twice a week to ensure lasting ROM and sustained beneficial effects.

When done prior to exercise, PNF has been shown to decrease muscle strength, power, EMG activity, vertical jump height, and ground reaction time (Bradley et al., 2007; Marek et al., 2005; Mikolajec et al., 2012). This may be due to the muscles being stretched too far outside of their capacity, causing inhibition following the stretching. However, PNF has been shown to be beneficial for submaximal exercises such as jogging. Increased stride length, frequency, and ROM were recorded by Caplan et al. (2009) in 18 professional rugby players jogging at 80% of maximal effort over a five week period. Nelson et al. (2005) found PNF to be even more beneficial than strength training in increasing strength and athletic performance in untrained individuals over an 8 week period; muscle power, strength, and ROM increased during the protocol. Therefore, PNF stretching should be completed after exercise at least two times a week to increase ROM and induce increases in muscle strength, power, and athletic performance. PNF exercises done before exercise will diminish performance for the short term (90 minutes), however the long term effects may be similar (Funk et al., 2003).

### Effects on ROM

Funk et al. (2003) assessed the efficacy of PNF stretching versus static stretching on hamstring flexibility performed with or without exercise in a study of 40 undergraduate student-athletes. Each stretching method was performed for five minutes after 60 minutes of exercise or no exercise. The results showed that those who exercised and received PNF stretching experienced more of an increase in flexibility when compared to the baseline group and the group without exercise and PNF. However, there were no differences observed in the static stretching groups (baseline, with exercise, and without exercise).

Lucas and Koslow (1984) recruited 63 college women for their seven week study in which they examined the effects of three methods of stretching on the hamstring and gastrocnemius muscles. These three stretching techniques included static, dynamic, and the CR method of PNF. Each subject was assigned to one of the three treatment groups and received three treatments a week. Three measuring ROM tests were performed on all subjects; before the treatments began, after 11 rounds of treatment, and after all 21 rounds of treatment had been completed. Each of the treatments was found to produce significant improvements when comparing the beginning test to the end test. It turned out that the longer the treatment time, the less significant the results differed among the three treatments.

Wallin et al. (1985) performed a study on 47 male subjects who were randomly assigned to four treatment groups. These four groups represented each group of TMs being stretched; the gastrocnemius, the ankle dorsiflexors, the hip adductors, or the hamstrings. The gastrocnemius, hamstring, and adductor groups received 14 treatment bouts of the CR method of PNF, while the ankle dorsiflexor group received a BS method. The ankle dorsiflexor group was switched to the CR method afterwards. Flexibility was increased more with the CR method than with the BS method for this group.

Etnyre and Lee (1988) assessed 74 subjects, 49 men and 25 women, in order to compare changes in hip flexion and shoulder extension between men and women through SS, CR, and CRAC stretching techniques, over 12 weeks. ROM measurements were obtained from all subjects before any treatment began and were taken once every three weeks until the end of the study. Significant increases in ROM were seen throughout the treatment groups, but it was found that the PNF techniques were more effective than the SS method for both hip flexion and shoulder extension.

Women generally started off with greater ROM in both of the movements being studied, though the results proved that the increases that the men and women made were not significantly different when compared to one another. According to the results though, men had greater increases with the CRAC method than they did with the CR method. Women differed from the men in that they did not have very significant ROM increase differences between either PNF method at either joint.

Feland et al. (2001) investigated 97 randomly selected elderly athletes to study the changes in flexibility of the hamstrings after stretching prior to exercise in an elderly population. The subjects were assigned to one of three groups: control, the CR method, or the SS method. No significant differences between the SS and CR treatment groups were found, though the differences determined were more pronounced in the men compared to the women. These differences between genders were even more pronounced amongst the younger subjects. As it turns out, age affects the flexibility gains in the CR method. As age increases, the soft tissues that are usually affected by PNF methods and receive the neural inhibition produced by PNF to reduce reflex activity and promote relaxation, which leads to greater ROM, are changed. The soft-tissue matrices tend to lose elasticity and strength, and myofibrils are replaced by connective tissue. These changes cause the older muscles to be more susceptible to contraction-induced injury (Feland et al., 2001).

Feland and Marin (2004) assessed 72 subjects to determine if submaximal contractions during the CR method of PNF on the hamstrings would yield comparable flexibility gains to MVICs. 60 of the subjects were randomly put into one of the three treatment groups, which included 20% of MVIC, 60% of MVIC, and 100% of MVIC, while the 12 remaining were put in the control group. Each subject in the first three groups performed three six second CR method stretches, all at their respective intensities, with 10 second breaks in between each contraction for five days. The results showed that contractions at 20% and 60% of MVIC are just as effective as 100% of MVIC during the CR method of PNF because they all increased flexibility.

Rowlands et al. (2003) recruited 43 subjects to investigate the effect of varying contraction durations during PNF stretching on increased flexion ROM at the hip. Each subject was assigned to one of three groups. These groups included five second isometric contractions, ten second isometric contractions, and the control. The two treatment groups performed the CRAC method of PNF two times per week, for six total weeks, with at least 24 hours between the two weekly treatments. They performed a five minute warm-up, a five minute SS, and then two kinds of CRAC method PNF stretches three times each. For both methods, the subjects held the isometric contraction of the hamstrings for their respective time. Significant increases were noted for both treatment groups after three weeks and six treatments of CRAC method of PNF. Even more improvement was found after six weeks and twelve treatments. It was determined that the longer stretching time did produce greater flexion ROM increases for the subjects.

The results of these seven studies discussing ROM (436 subjects) imply that PNF, both the CR and the CRAC methods, increases ROM and flexibility in all of the subjects at any percentage of the MVIC. Increases were more significant when PNF methods were performed after exercising, and the longer the contraction was held by reducing contractile activity (Bonnar et al., 2004; Magnusson, 1998). However, this increase in flexibility and ROM is not permanent. Its effects were found to last for only six minutes after the stretching protocol ended (Spernoga et al., 2011). In order to maintain it, performing PNF over a longer period of time is required, although the results become less significant the longer the treatment time is, and the more it is performed over a longer period of time. There is a very significant increase after the first bout of treatment, therefore PNF is a good way to gain immediate improvements in ROM of a joint. PNF methods increase the flexibility and ROM of all subjects who received PNF stretching, but there are some differences between gender and age groups. It was discovered that men had more increases in flexibility and ROM with the CRAC method than women did (Etnyre and Lee, 1988). This difference between genders holds true even with different age groups. As the subjects got older though, it was discovered that there were fewer differences in flexibility and ROM gains found before and after the PNF methods. Because there is a higher probability that older people will get injured from the intense contraction during PNF, this decrease in differences could possibly mean that PNF methods should not be utilized on the elderly (Feland et al., 2001).

## Conclusion

Research indicates that PNF stretching, both the CR and CRAC methods, are effective in improving and maintaining ROM, increasing muscular strength and power, and increasing athletic performance, especially after exercise. However, proper protocol and consistency must be followed to attain and maintain the benefits of PNF techniques. Four theoretical mechanisms were proposed as being responsible for these benefits, although there is little empirical evidence to support these mechanisms. Further research should be completed to prove the efficacy of each of these mechanisms in the factors affected by PNF
